# Regulatory Mechanism of Opposite Charges on Chiral Self-Assembly of Cellulose Nanocrystals

**DOI:** 10.3390/molecules28041857

**Published:** 2023-02-15

**Authors:** Bin Wang, Jinyang Xu, Chengliang Duan, Jinpeng Li, Jinsong Zeng, Jun Xu, Wenhua Gao, Kefu Chen

**Affiliations:** 1 Plant Fiber Material Science Research Center, State Key Laboratory of Pulp and Paper Engineering, School of Light Industry and Engineering, South China University of Technology, Guangzhou 510640, China; 2 Guangdong Provincial Key Laboratory of Plant Resources Biorefinery, Guangzhou 510006, China

**Keywords:** cellulose nanocrystals, chiral nematic liquid crystal, charge regulation, chiral self-assembly, structural color

## Abstract

The charge plays an important role in cellulose nanocrystal (CNC) self-assembly to form liquid crystal structures, which has rarely been systematically explored. In this work, a novel technique combining atomic force microscopy force and atomistic molecular dynamics simulations was addressed for the first time to systematically investigate the differences in the CNC self-assembly caused by external positive and negative charges at the microscopic level, wherein sodium polyacrylate (PAAS) and chitosan oligosaccharides (COS) were used as external positive and negative charge additives, respectively. The results show that although the two additives both make the color of CNC films shift blue and eventually disappear, their regulatory mechanisms are, respectively, related to the extrusion of CNC particles by PAAS and the reduction in CNC surface charge by COS. The two effects both decreased the spacing between CNC particles and further increased the cross angle of CNC stacking arrangement, which finally led to the color variations. Moreover, the disappearance of color was proved to be due to the kinetic arrest of CNC suspensions before forming chiral nematic structure with the addition of PAAS and COS. This work provides an updated theoretical basis for the detailed disclosure of the CNC self-assembly mechanism.

## 1. Introduction

In nature, many creatures have stronger viability due to their intrinsic structural colors, such as peacocks using colored plumes to court and insects using the color of back shells to camouflage themselves from natural enemies [1]. Different from the pigment colorations, these structural colors arise from the interaction between ordered chiral nematic structures in organisms and light. Because of the advantages of higher color saturation and never fading, these spiral structures contribute to manipulating light and color. Cellulose nanocrystal (CNC) is a kind of rod-like nanoparticle commonly derived from natural cellulose raw materials via sulfuric acid hydrolysis, which can construct layered chiral nematic structures and display typical lyotropic liquid crystal phenomena by evaporation-induced self-assembly (EISA). In this context, the self-assembly of cellulose nanocrystals (CNCs) into structurally colored films has attracted considerable interest in the scientific community and beyond as a potential candidate to produce more sustainable photonic pigment since they were first discovered in 1959 [2].

To better utilize CNC liquid crystal materials, it is significant to reveal the self-assembly trajectory of CNC particles and the formation mechanism of CNC chiral helical structures at the sub-micro level. Initially, Revol and Marchessault speculated that the CNC chiral structure was induced by its spirally twisted morphology [3]. After that, Orts et al. and Ogawa confirmed the twisted morphology of CNCs by using small-angle neutron diffraction and electron microdiffraction combined with cryo-transmission electron microscopy, respectively [4,5]. In addition, Araki et al. found that bacterial cellulose microcrystals could exhibit chiral nematic structures with the presence of electrolytes, while the chiral structures disappeared in the absence of electrolytes [6]. This phenomenon was attributed to the ability of the electrolyte to shrink the effective particle size and thus make the intrinsic twists of the BC more effective, which indicated that the precise regulation of the twist structures was essential for the formation of CNCs into chiral liquid crystal orders. Moreover, the Onsager theory proposed that the concentration of uncharged rigid rod-like particles forming an ordered liquid crystal phase was only related to the aspect ratio of the particles [7]. However, for negatively charged particles like CNCs, the Onsager model was not fully applicable because of the electrostatic force, which would have a drastic effect on the free energy of the whole system. Subsequently, to further investigate the principle of the phase separation process of charged rod-like nanoparticles, the Onsager model was modified by introducing two parameters to characterize the effect of electrostatic interactions [8]. Through this research, the importance of charge in the formation of CNC chiral structures became self-evident, and recently, a larger body of work has been dedicated to chiral assemblies. For example, Abitbol et al. found that the reduction in surface charge accelerated the phase separation process and created relatively long-spaced ordered phases, which was attributed to a decrease in available free volume and the screening of particle shape by the extended electrostatic double layers [9]. Furthermore, Dong et al. found that the chiral nematic pitch of the anisotropic phase decreased with increasing electrolyte concentration due to the decrease in the double layer thickness [10]. Although these phenomena seem to indicate that the twisted morphology and surface charge of CNC affect the formation of chiral structures, the mechanism still needs to be further studied. Therefore, it is essential to uncover the principles for the promotion of the self-assembly of CNCs with different charges into chiral structures, especially for the CNC behavior trajectory at the micro scale.

Herein, sodium polyacrylate (PAAS) and chitosan oligosaccharide (COS) carrying opposite charges were introduced into the CNC suspension system to investigate the effect of opposite charges on the CNC self-assembly behavior in EISA. We found that the structural color of the resultant composite films presented a blue-shift and even disappearance, though PAAS and COS exhibit opposite charge properties. To systematically interpret the phenomenon, the associative techniques of quartz crystal microbalance (QCM), atomic force microscopy (AFM) force tests, and atomistic molecular dynamics (MD) simulations were employed to quantify intermolecular forces between the twist profile CNCs and the charge-regulating media, which were further used to determine the cross angle of CNC stacking as a function of distance and further understand the mechanism of the color’s blue-shift. Moreover, the phase separation experiments and rheology tests were introduced to explain the self-assembly trajectory of CNC particles. Finally, this work clarifies the role of external charges in CNC suspensions during EISA and explains the reasons for the blue-shift and eventual disappearance of structural colors, providing theoretical guidance for the design and application of CNC-derived chiral optics materials.

## 2. Results and Discussion

### 2.1. Adjustment of CNC Photonic Films by PAAS and COS

In this work, the rod-like CNCs prepared by the traditional sulfuric acid method possessed an average height and length of 9.3 nm and 184.2 nm, respectively (Figure S1). In the process of EISA, the uniform dispersion of the CNCs is the prerequisite for the orderly formation of CNC chiral nematic structure, which depends on the charge intensity of the CNCs. Subsequently, the zeta potential (ζ-potential) of the CNC suspension was determined to be −37.7 mV and CNC suspensions exhibited a clear Tyndall effect (Figure S2), indicating that a stable colloidal suspension can be formed [11].

In order to avoid other interference factors, we used PAAS and COS as the charge control source in the CNC system to fully investigate the effect of external charges on the CNC chiral nematic structure. Due to the highly negatively charged density of PAAS (ζ-potential of approximately −56 mV), it prevented PAAS from adsorbing with CNCs. Meanwhile, the absolute value of ζ-potential of the mixtures increased with the increasing addition of PPAS (Figure S2a). Unlike PAAS, COS is positively charged based on its amino group (ζ-potential of +10.0 mV), which enables the adsorption of COS onto CNCs and decreases the absolute ζ-potential value of the CNC/COS mixtures. Although the ζ-potential decreased to −26.5 mV as the COS was loaded with 3.0 wt% (Figure S2b), the CNC/COS mixtures could still maintain a stable dispersion system [11].

**Figure 1:**
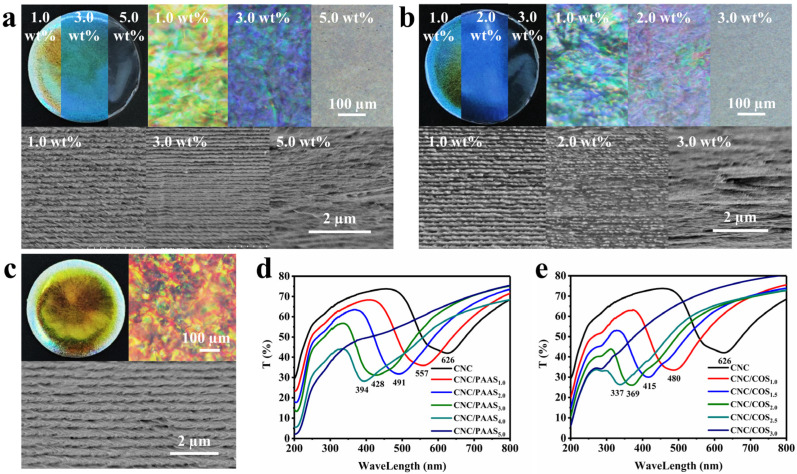
The optical photographs (**top left**), POM patterns (**top right**), and SEM images (**below**) of (**a**) CNC/PAAS films (the ratio of PAAS is 1.0 wt%, 3.0 wt%, and 5.0 wt%, respectively), (**b**) CNC/COS films (the ratio of COS is 1.0 wt%, 2.0 wt%, and 3.0 wt%, respectively), and (**c**) pure CNC film. The UV spectrums of (**d**) CNC/PAAS films and (**e**) CNC/COS films.

Figure 1a–c and Figure S3 show the polarized optical microscope (POM) patterns in reflectance mode for all films. Digitalized colors were presented within the same film under crossed polarizers, resulting from a discrepancy in the cholesteric pitch and potentially a change in the tilt of the helical axis within different areas of the film [12]. In addition, the single macroscopic dominating color of the films changed with the addition of polymers, proving that PAAS and COS can orderly regulate the optical signal of CNC liquid crystal film. More interestingly, unlike previous studies on the red-shift of the structural color of CNC films caused by polymer [13], the addition of PAAS or COS resulted in a continuous blue-shift of structural color of the CNC films. As shown in Figure 1d,e, the photonic bandgap (PBG) wavelengths of the CNC/PAAS films decreased from 626 nm to 557 nm, 491 nm, 428 nm, and 394 nm, and finally the peak disappeared as the fraction of PAAS increased from 0 to 5.0 wt% in sequence, accompanied by a continuous blue-shift of the structural color from red to purple, and even colorless (Figure 1a). Similarly, the PBG wavelengths of the CNC/COS films decreased sequentially from 626 nm to 480 nm, 415 nm, 369 nm, and 337 nm, and finally the peak disappeared, correlating with the structural color change of the film (Figure 1b). The striking phenomenon that this opposite charge change trend induces the same macroscopic structure color change has attracted our attention. 

### 2.2. The Mechanism of Blue-Shift of CNC Structural Color

To find the origin of blue-shift of structure color, the microstructures of CNC/PAAS and CNC/COS films were observed by scanning electron microscopy (SEM) (Figure 1a–c). The parallel layer microstructure remaining in the films indicated the pitch length and the preferential orientation of CNCs (handedness and direction) [12]. With the addition of polymers, the pitch of the films gradually decreased, which enabled the blue-shift of structural color. It can be proved by the following formula:

(1) λ=n·P·sinθ

where *λ* represents the PBG wavelength, *n* is the average refractive index of the CNC film, *P* is the helical period, and *θ* is the angle of reflection of light on a film. Under the premise that the *n* and *θ* are constant, the decrease in *P* leads to the decrease in PBG wavelength. To further clarify the mechanism of pitch change, the roles of PAAS and COS in the CNC self-assembly process were further investigated at the micro scale.

#### 2.2.1. Interaction between Particles in Aqueous Solutions 

**Figure 2:**
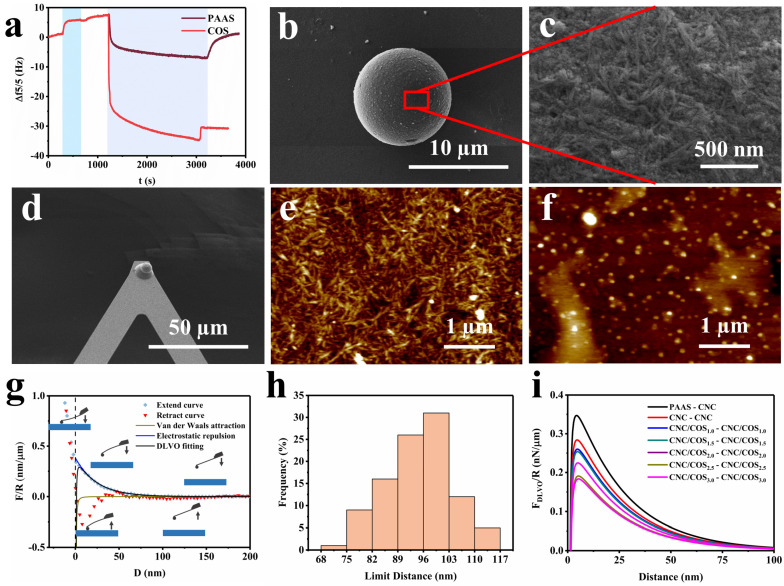
(**a**) QCM−D studies of PAAS and COS adsorption on a CNC−coated sensor surface. (**b**) SEM image and (**c**) enlarged view of the CNC−modified silica microsphere. (**d**) SEM image of a modified AFM probe equipped with a SiO_2_ microsphere (diameter of 10μm). AFM images of (**e**) CNC−coated substrate and (**f**) PAAS−coated substrate, respectively. (**g**) The force−distance curves and the DLVO fitting curves of CNC−CNC and schematic diagram of the AFM force testing process. (**h**) The limit distance of the interaction of CNC−CNC. (**i**) The DLVO re−fitting curves.

Firstly, quartz crystal microbalance with dissipation (QCM-D) measurements were used to quantitatively evaluate the adsorption of COS or PAAS on the CNC surface to understand the distribution behavior of polymers in CNC suspensions. The adsorption curves are shown in Figure 2a. In the initial stage of the test, a 0.1 wt% CNC suspension was injected to stabilize the CNC-coated sensor followed by DI water until a stable baseline was obtained. PAAS or COS was then injected for adsorption behavior analysis. When PAAS was injected, the curve showed a shallow trough, suggesting a small amount of sorption on the sensor. Surprisingly, the adsorption mass calculated by Sauerbrey equation [14] finally dropped to 76 ng/cm^2^ after DI water rinsing to equilibrium. This was attributed to spatial resistance rather than to the presence of adsorption between PAAS and CNCs. Obviously, COS could be firmly attached to the CNC surface because of the electrostatic attraction, and the final adsorption mass was stabilized at 676 ng/cm^2^. Depending on the diameter of the quartz crystal and the CNC amount applied, the adsorption amount was converted to 87 mg/g_CNC_ for COS and 10 mg/g_CNC_ for PAAS. The results of ζ-potential and QCM-D measurements suggested that PAAS was dissociated from the CNCs, while COS adsorbed completely on the CNC surface. Therefore, we perform the AFM force measurement to measure the interactions among CNCs, PAAS, and CNC/COS_j_ (j = 1.0, 1.5, 2.0, 2.5, and 3.0, respectively) particles.

Prior to AFM force testing, the probes and substrates needed to be modified. As shown in the SEM images (Figure 2b–d, Figure S4a–c), the samples were adsorbed on a surface of the uniform-sized amino silica microspheres and the modified silica micro-sphere was tightly adhered onto an AFM tipless probe. The AFM images (Figure 2e,f, Figure S4d) also illustrated that the substrates were uniformly coated with a layer of samples. For the force curve, it can be divided into two parts, the extend curve and the retract curve, corresponding to the process of the probe approaching the substrate and then moving away (Figure 2g). To avoid the influence of the adhesion force between the probe and the substrate, the approaching part of the force curve was chosen for analysis. In addition, the classical Derjaguin−Landau, Verwey−Overbeek (DLVO) theory was adopted to analyze the force curves [15]. This theory suggests that the interaction energy between two colloidal particles is the sum of the van der Waals (VDW) attraction interaction and the electrostatic double layer (EDL) repulsive interaction [16]. The DLVO force (*F_DLVO_*), the VDW force (*F_VDW_*), and the EDL force (*F_EDL_*) as a function of distance present the relationship shown in Equation (2):

(2) $$F_{DLVO}\left(D\right)=F_{VDW}\left(D\right)+F_{EDL}\left(D\right)$$

The interactions between the tip and the substrate for different geometries are summarized by Israelachvili [17]. The calculation formulas of the EDL force and the VDW force based on a sphere–plate model are shown as follows:

(3) $$F_{EDL}\left(D\right)=\kappa rZe^{-\kappa D}$$

(4) $$\kappa =\sqrt{\left(\frac{2F^{2}c_{0}}{\varepsilon \varepsilon_{0}RT}\right)}$$

(5) $$Z=\frac{64\pi \varepsilon \varepsilon_{0}R^{2}T^{2}}{F^{2}}\tan h^{2}\left(\frac{F\psi_{\delta }}{4RT}\right)$$

where *κ* is the inverse of the Debye constant. *Z* is the interaction constant of the electrostatic repulsive force, *r* is the radius of the SiO_2_ sphere (*r* = 5 µm), *D* is the distance between probe and substrate, *F* is the Faraday constant, *R* is the gas constants, *c*_0_ is the electrolyte concentration of the medium, *ε*_0_ is the vacuum permittivity, and *ε* is the relative permittivity of water. *T*, *h*, and *ψ_δ_* are the temperature, Planck constant, and Stern layer potential, respectively.

(6) $$F_{VDW}\left(D\right)=-\frac{A_{H}\cdot r}{6D^{2}}$$

(7) $$\begin{array}{c} A_{H}=\frac{3}{4}kT\left(\frac{\varepsilon_{1}-\varepsilon_{3}}{\varepsilon_{1}+\varepsilon_{3}}\right)\left(\frac{\varepsilon_{2}-\varepsilon_{3}}{\varepsilon_{2}+\varepsilon_{3}}\right)+\\ 3\frac{hv_{e}}{8\sqrt{2}}\frac{\left(n_{1}^{2}-n_{3}^{2}\right)\left(n_{2}^{2}-n_{3}^{2}\right)}{{\left(n_{1}^{2}+n_{3}^{2}\right)}^{\frac{1}{2}}{\left(n_{2}^{2}+n_{3}^{2}\right)}^{\frac{1}{2}}\left[{\left(n_{1}^{2}+n_{3}^{2}\right)}^{\frac{1}{2}}+{\left(n_{2}^{2}+n_{3}^{2}\right)}^{\frac{1}{2}}\right]} \end{array}$$

where *A_H_* is the Hamaker constant, estimated to be 3.6 × 10^−21^ J for CNCs by Cao et al. [15]. *ε*_1_, *ε*_2_, *ε*_3_ represent the static dielectric constants of the sample on the probe, sample on the substrate, and water, respectively. *n*_1_, *n*_2_, *n*_3_ represent the refractive index of the three media at the wavelength of visible light, respectively. *υ_e_* is the main electronic absorption frequency in the ultraviolet (UV) region. *k* is the Boltzmann constant.

In general, under a constant external environmental condition, *κ* is a constant value and *Z* is related to *ψ_δ_* only. Therefore, the *F_EDL_* is affected by the distance and the potential of the colloidal particles (Equations (3)–(5)). The *F_VDW_* is only a function of the distance (D) and is so small that it can be negligible at large distances (Equations (6) and (7)). In this case, it can be assumed that the *F_EDL_* occupies all parts of the *F_DLVO_* at large distances. Since the *F_EDL_* increases exponentially with the distance, the force curves were subjected to a natural logarithmic transformation followed by a linear fit at larger distance. Then, *κ* and *Z* were calculated from the slope and intercept of the fitted line. Finally, the constants were brought into the equations to obtain the DLVO fitting curve.

The force curves and the DLVO fitting curves are shown in Figure 2g and Figure S5, and the average values of constant *κ* and constant *Z* for different groups of samples are listed in Table 1. Clearly, the DLVO fitting curves fitted well with the force curves from the tests at medium and large distances. A tiny discrepancy between the fitted curves and the actual curves at the close distance can be found, which was because of the spatial resistance and the adsorption behavior occurring instantaneously between the probe and the substrate. Since these nanoparticles were able to interact with each other at the range of medium to long distances in suspensions, we believed that the existing discrepancy had little effect on the fitting results. As presented in Table 1, the value of *Z* decreased as the absolute value of the ζ-potential of the sample on the probe and the substrate decreased, which was in accordance with Equation (5). However, the *κ* value, that theoretically does not change, showed roughly an increasing trend. We speculated that the particles were released into the water during submerged measurement, which caused the changes in electrolyte concentration (*c*_0_). As the sample charge and the adsorption stability decreased, the growing sample particles were released into the water from the SiO_2_ microsphere and substrate and raised the electrolyte concentration of the system, leading to an increase in the *κ* value. In fact, the amounts of polymers added in this work were so small that they had almost no effect on the electrolyte concentration of the CNC suspension. To ensure the reliability of the fitting results, the *κ* value of 0.04125 nm^−1^ calculated from the CNC-CNC test was fixed to re-fit all the curves. The DLVO re-fitting curves are shown in Figure 2i. It is clear that the *F_DLVO_* between PAAS and CNCs was greater than that of the force between CNCs, and the *F_DLVO_* between CNC/COS composite particles gradually decreased as the ratio of COS increased.

In addition, the limit distance of the interaction for different groups of samples was further counted according to the DLVO curves (Figure 2h, Figure S6). The limit distance was determined as the point where the slope of the curve shifted to 0 and referred to the distance between particles in an equilibrium suspension system. When the distance between particles was less than the limit distance, it was in the repulsive force regime; the existing repulsive force allowed the particles to move away from each other. During the self-assembly process, the particles approaching each other would stabilize at the limit distance where the combined external force is near 0. The average limit distances of each sample group are listed in Table 1. Obviously, the limit distances between PAAS particles and/or PAAS and CNCs were greater than those of the CNC particles because of the coulombic interaction, suggesting that the PAAS will inevitably squeeze the space of CNCs in the EISA process. Since PAAS tended to converge outside the crystallites instead of being dispersed between CNCs [18], the compression of CNCs by PAAS led to a reduction in the spacing of CNCs. Similar to adding PAAS, the limit distance between CNC/COS composite particles also decreased with the addition of COS (Table 1). The reason was that COS adsorbed on the CNC surface to form composite particles, and the charge of the CNC/COS composite particles decreased as the COS ratio increased.

#### 2.2.2. Analysis of Twisting and Approach Process of CNCs

It is already clear that the addition of PAAS and COS will lead to a reduction in the distance between CNCs in suspension. However, the subsequent effect of the reduction in the spacing of CNCs during the self-assembly process remains an issue. Therefore, MD simulations were used to investigate the CNC morphology and concrete evolutionary process of these particles in water.

**Figure 3:**
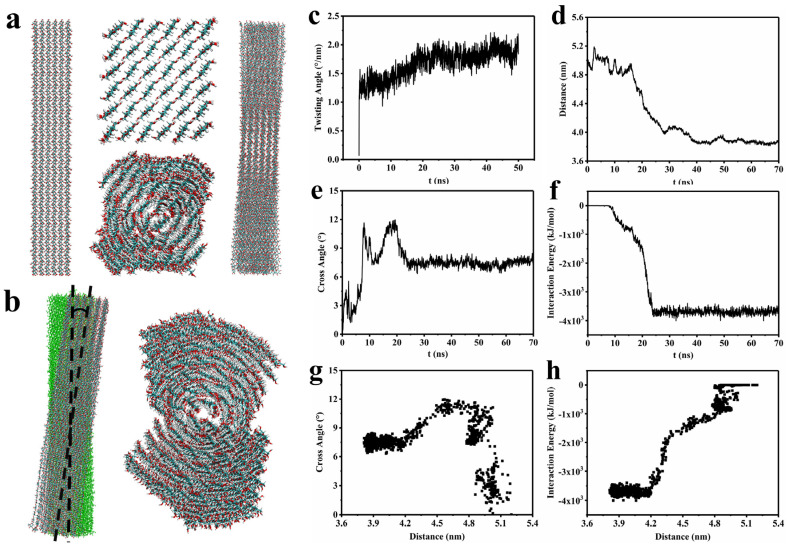
(**a**) Side views (**left**) and cross−sections (**upper middle**) of the initial structure and side views (**right**) and cross−sections (**lower middle**) of the twist structure. (**b**) Side view (**left**) and top cross−section (**right**) of the dimer system; the angle between the long axes of the two models was defined as the cross angle. The curves of (**c**) twisting angle, (**d**) distance, (**e**) cross angle, and (**f**) interaction energy vs. simulation time, respectively. (**g**) The relationships between cross angle and distance. (**h**) The relationships between interaction energy and distance.

Considering the effect of twist morphology on the self-assembly process, it is necessary to first model the twisted CNCs to ensure the accuracy of the simulation approach process. The initial structure of the constructed CNCs comprised 6×6 parallel glucose chains with 40 repeating units, the long axis of which was oriented along the *Z*-axis. Significantly, the final twist structure of CNCs was produced after equilibration runs of 5 ns and production runs of 50 ns for the initial structure, which was mainly due to the orientation of the chains in opposite directions of the fiber cross-section instead of the bending of individual chains [19]. Figure 3a shows side views and cross-sections of the initial structure and the twist structure. The final size of the CNC model was tested to be 3.5 nm × 3.7 nm × 21.1 nm, similar to the average size of crystalline regions in commercial nanocellulose. The twisting degree, characterized by an angle between the orientations of the chains on the opposite sides of a fibril, was ultimately stabilized at approximately 2°/nm (Figure 3c), which was consistent with the result of Paavilainen et al. [19].

Subsequently, the CNC model was replicated in the × direction for the nanocrystal dimer simulation, which was used for the investigation of the relationship between distance and cross angle (Figure 3b). During this investigation, one nanocrystal of the dimer was moved for a certain distance and rotated for a small angle to check the energy change. The variation of the dimer structure during the 70 ns evolution is illustrated in Figures S7 and S8. In the aqueous system, the two nanocrystals in the dimer gradually approached and finally stabilized at a barycenter distance of 3.8 nm under the VDW attraction and hydrogen bonding forces in close proximity (Figure 3d). It is worth noting that the dimer eventually exhibited a stacking arrangement with a certain cross angle as the two nanocrystals approached among the twisted rod-like CNC structure (Figure 3b). As presented in Figure 3e,f, the cross angle reached about 7 degrees after 30 ns, while the adsorption energy reached its maximum simultaneously, meaning that the whole dimer system was at equilibrium in this state. In addition, the variations of the cross angle with distance and the interaction energy with distance were summarized in Figure 3g,h, respectively. It was obvious that the cross angle increased overall with the proximity of the two nanocrystals, except at a spacing of 4.8 nm, where the crossover angle suddenly increased to 12 degrees due to the instability of the system caused by solvent collisions.

Combined with the result of the AFM force test, it was found that the equilibrium distance of CNCs in aqueous suspension decreased with the addition of PAAS and COS, while the cross angle of CNCs in a stacking arrangement increased with the addition of PAAS, resulting from the squeezing effect of PAAS on CNCs in the CNC/PAAS mixtures. Moreover, the cross angle also increased with the addition of COS, which was attributed to the decrease in the equilibrium distance caused by the ζ-potential changes. The increase in the cross angle led to a decrease in the number of CNC layers for the formation of a whole helical period (360°); therefore, the decreased pitch of the CNC photonic crystal simultaneously caused the blue-shift of the CNC films after the addition of PAAS or COS.

### 2.3. The Mechanism of Disappearance of Structural Color

With the ratio of PAAS and COS increasing to 5.0 wt% and 3.0 wt%, respectively, the structural color of CNC/PAAS and CNC/COS films tended to be colorless. Initially, we thought that this phenomenon originated from the movement of structure color to the invisible light region with the increase in PAAS and COS. However, the chiral collapse structure in SEM images and the absence of birefringence in POM images negated this assumption (Figure 1a,b), opening up another possibility related to phase separation and gelation behavior [20]. To confirm the origin of the disappearance of structural color, phase separation experiments and rheology tests were performed.

**Figure 4:**
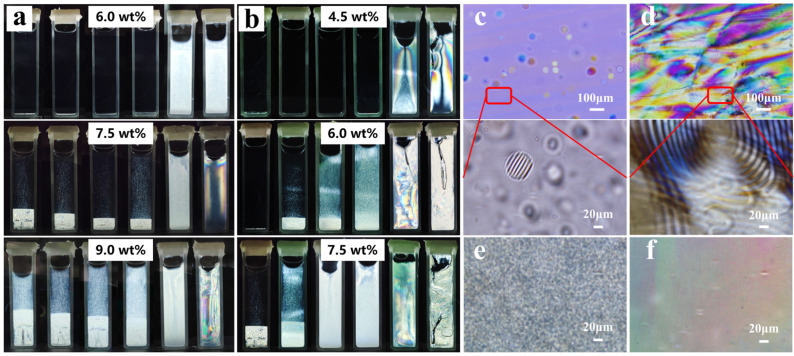
(**a**) Photos of CNC/PAAS mixtures taken between crossed linear polarizers at different CNC concentrations (6.0 wt%, 7.5 wt%, and 9.0 wt%, respectively); the ratio of PAAS is 0 wt%, 1.0 wt%, 2.0 wt%, 3.0 wt%, 4.0 wt%, and 5.0 wt% in order, from left to right. (**b**) Photos of CNC/COS mixtures at different CNC concentrations (4.5 wt%, 6.0 wt%, and 7.5 wt%, respectively); the ratio of COS is 0 wt%, 1.0 wt%, 1.5 wt%, 2.0 wt%, 2.5 wt%, and 3.0 wt% in order, from left to right. (**c**) The POM patterns of the isotropic phase after phase separation (**top**) and its partial enlarged view (**below**). (**d**) The POM patterns of the anisotropic phase (**top**) and its partial enlarged view (**below**). The POM patterns of (**e**) CNC/PAAS_4.0_ mixture and (**f**) CNC/PAAS_5.0_ mixture.

To simulate the evaporation process of suspensions, the CNC suspension (9.0 wt%) was diluted to different concentrations in sealed cuvettes for the phase separation monitoring. After three more weeks in suspension, the cuvettes were viewed between crossed linear polarizers (Figure 4a,b). For the pure CNC suspensions, the 6.0 wt% suspensions still remained essentially isotropic, whereas the phase separation occurred at 7.5 wt%, and the percentage of anisotropic phase increased with further concentration. The anisotropic phase shows a bright birefringent pattern in the lower layer of the suspensions, while the dark isotropic phase is in the upper layer [21]. When PAAS was added to the suspension, the percentage of anisotropic phase decreased. Evidently, the concentration at which the anisotropic phase first appeared (C_a_) had little change when the PAAS addition was increased from 1.0 wt% to 3.0 wt%. Surprisingly, the CNC/PAAS mixtures presented total birefringence at each concentration when the PAAS addition amount reached 4.0 wt%, representing the concentration at which the anisotropic phase was complete (C_i_), reduced to less than 6.0 wt%. For the CNC/COS mixtures, the percentage of anisotropic phase increased with the addition of COS, and the concentration C_a_ was decreased from 7.5 wt% to 6.0 wt% at low addition levels. Similarly, the concentration C_i_ dropped to below 4.5 wt% when the COS addition reached 2.5 wt%.

Then, POM was performed on the phase-separated samples. In the isotropic phase, spherical tactoids with short-range-ordered structures were observed (Figure 4c) as an intermediate state bridging the isotropic phase and the chiral nematic liquid crystalline phase with long-range anisotropy (Figure 4d) [22]. In addition, we found that the CNC/PAAS_4.0_ suspension showed fingerprint patterns (Figure 4e), while the CNC/PAAS_5.0_ suspension showed only colored birefringent domains (Figure 4f), although they were both perfectly bright under the observation of the crossed linear polarizers. In addition, this phenomenon was also observed in the CNC/COS suspensions, indicating that the suspension only formed a nematic liquid crystal without a long-range chiral structure rather than a chiral nematic liquid crystal with the addition of 3.0 wt% COS and 5.0 wt% PAAS [6].

The variations of viscosity and gelation behavior can also affect the self-assembly process when external polymers are added [20]. Therefore, the steady shear viscosity measurements and oscillatory rheological experiments were performed for CNC composite systems at a range of concentrations. 

**Figure 5:**
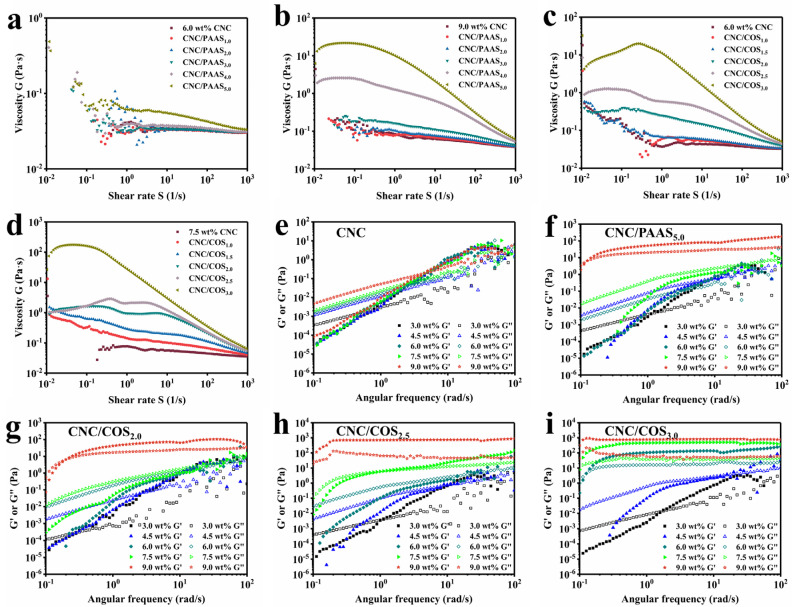
The steady viscosity of CNC/PAAS mixtures at (**a**) 6.0 wt% and (**b**) 9.0 wt% concentration, respectively. The steady viscosity of CNC/COS mixtures at (**c**) 6.0 wt% and (**d**) 7.5 wt% concentration, respectively. Dependence of storage modulus (G′) and loss modulus (G″) as a function of the angular frequency for (**e**) CNC suspension, (**f**) CNC/PAAS mixtures with 5.0 wt% PAAS addition, and CNC/COS mixtures with (**g**) 2.0 wt%, (**h**) 2.5 wt%, and (**i**) 3.0 wt% COS addition.

The steady viscosities for the composite suspensions at different concentrations are shown in Figure 5a–d and Figure S9. All the viscosity curves exhibited a low shear rate Newtonian plateau followed by a typical shear-thinning region related to the shear alignment of individual CNC particles [9]. The steady viscosity of all samples increased with the increase in suspension concentration and polymer addition at low to medium shear rates, whereas the viscosity of different samples would approach each other at a relatively high shear rate. The increase in viscosity with concentration was attributed to the large excluded volume required for “tumbling” the rod-shaped crystals around their length due to shear-induced orientation [23]. As anionic polymerization chains of small molecules, PAAS tended to move outside crystallites where electrostatic repulsion was not predominant rather than to interleave between negatively charged CNCs [18], which compressed the space occupied by CNCs and made them difficult to “tumble”, similar to the case of increasing concentration. Therefore, the addition of PAAS increased the viscosity of the suspensions (Figure 5a,b). Due to the positively charged amino group, COS reduced the surface charge of CNCs after adsorption on CNCs, which induced the CNCs’ flocculation and thus increased the viscosity of the samples (Figure 5c,d) [23]. Moreover, the viscosity curve of the 6.0 wt% CNC/PAAS_5.0_ mixture showed two shear-thinning regions at low and high shear rates separated by a region of very weak thinning, which is typical in liquid crystal (LC) suspensions (Figure 5a). The other viscosity curves of the CNC/PAAS mixtures at 9.0 wt% concentration showed the similar typical regions of LC suspensions (Figure 5b). This phenomenon occurred at lower concentrations for COS-added suspensions compared to PAAS. The viscosity curves of the CNC/COS_3.0_ mixture and others exhibited the typical trend in LC suspensions at concentrations of 4.5 wt% and 7.5 wt% (Figure 5c,d), respectively, which was well consistent with the results of phase separation experiments.

The oscillatory frequency sweep was executed to clarify the viscoelastic properties of the composite suspensions. The correlations between modulus (G′ and G″) and angular frequency ω are shown in Figure 5e–i and Figure S10. For the CNC/PAAS_5.0_ mixture with concentration of 9.0 wt% (Figure 5f), G′ was higher than G″ in the whole angular frequency range, indicating the rigid gel behavior, while other CNC/PAAS mixtures and the CNC suspension showed viscous rheological behavior (G″ > G′) at low angular frequency, displaying elastic rheological behavior (G″ < G′) at high angular frequency (Figure 5e, Figure S10a–d). The CNC/COS mixtures were more likely to form rigid gels because of the positively charged groups of COS. The CNC/COS_3.0_ mixture (Figure 5i) showed the gel-like behavior at 6.0 wt%, while the CNC/COS_2.5_ and CNC/COS_2.0_ mixtures occurred at 7.5 wt% and 9.0 wt% (Figure 5g,h), respectively. The transition of suspensions from liquid-like to gel-like state indicates that the system reaches kinetic arrest, which causes the freezing-in of the structure. If gelation occurs at a concentration below the critical concentration C_a_, the suspension will remain in the isotropic phase instead of forming a chiral nematic liquid crystal structure, resulting in a film without a fingerprint pattern or even a birefringence pattern [20]. For the CNC suspensions with 5.0 wt% PAAS and 3.0 wt% COS, they maintain nematic liquid crystal structures at 9.0 wt% concentration, as phase separation experiments showed, while they reached kinetic arrest at 9.0 wt% and 6.0 wt% concentrations, respectively. That was the reason why they did not convert to chiral structures and eventually obtained colorless films by evaporation.

## 3. Experiment

### 3.1. Materials

Microcrystalline cellulose (MCC) with a length of 65 µm, sodium polyacrylate (PAAS, M_w_ = 3000–5000 g/mol), and chitosan oligosaccharide (COS, M_w_ = 2000 g/mol) were supplied by Macklin Biochemical Co., Ltd. (Shanghai, China). Sulfuric acid (H_2_SO_4_, 98 wt%) and hydrogen peroxide (H_2_O_2_, 30 wt%) were purchased from Dongzheng Chemical Glass Instrument Co., Ltd. (Guangzhou, China). Monodisperse amino silica microspheres with a particle size of 10 µm were obtained from Macklin Biochemical Co., Ltd. (Shanghai, China). Deionized (DI) water was used in all experiments.

### 3.2. Preparation of CNC Suspension

The CNC suspension was prepared by the classical sulfuric acid hydrolysis of MCC [21]. Briefly, MCC was hydrolyzed in a H_2_SO_4_ solution (10 mL for 1 g MCC) with vigorous stirring at 45 °C, which was terminated by diluting with 10 times as much DI water after 45 min. Next, the mixture was left to stand for 12 h and then the upper clear liquid was discarded. The resulting suspension was washed by centrifugation with DI water for 8 min at 8000 rpm, and subsequently purified using dialysis membranes (cutoff weight of 14,000 Da) in DI water for two weeks with daily exchange of the water. Finally, a CNC suspension with a solid content of around 3.0 wt% was obtained.

### 3.3. Preparation of CNC-Based Structural Color Films

CNC/PAAS mixtures were prepared by adding PAAS aqueous solution (50 wt%) to 3.0 wt% CNC suspension with the varied mass percentage of PAAS to CNCs of 1.0 wt%, 2.0 wt%, 3.0 wt%, 4.0 wt%, and 5.0 wt%, respectively (the sample name was abbreviated as CNC/PAAS_i_, i = 1.0, 2.0, 3.0, 4.0, and 5.0, respectively). The mixtures were then vigorously stirred to reduce agglomerates that may have been generated during the mixing process, followed by sonication for 1 min to further preserve the uniform dispersions. Then, the dispersions were cast into a Petri dish and dried at room temperature for 2–3 days to obtain the CNC/PAAS films. In addition, the CNC/COS films were also prepared in a similar way to that for CNC/PAAS films, while the additions of COS were 1.0 wt%, 1.5 wt%, 2.0 wt%, 2.5 wt%, and 3.0 wt%, respectively (the sample name was abbreviated as CNC/COS_j_, j = 1.0, 1.5, 2.0, 2.5, and 3.0, respectively).

### 3.4. Phase Separation Experiments

The CNC and all composite suspensions were concentrated to 9.0 wt% by a rotary evaporator. The 9.0 wt% suspensions were then diluted from 3.0 to 9.0 wt% with DI water in 20 mL vials. Prior to filling the cuvettes, the dilutions were sonicated in an ice bath for 2 min, followed by placing them into rectangular cuvettes (2 mm × 10 mm × 100 mm) sealed by an epoxy resin. They were left for 3 weeks at room temperature to observe the phase separation. The cuvettes were observed with a polarized optical microscope (POM, BX53M, Olympus, Japan) and photographed between crossed linear polarizers with light shining from the back. The percentage of the anisotropic phase was determined by dividing the height of the anisotropic phase by the total height of suspension in the cuvette.

### 3.5. AFM Force Curves Measurements

AFM force curves were measured on a Multimode AFM Nanoscope-VIII (Bruker, Santa Barbara, CA, USA) using PeakForce™ ScanAssyst mode in fluid. The force vs. distance curves and limit interaction distance between the probes and substrates were measured.

Prior to testing, the AFM probes and substrates were modified. Monodisperse amino silica microspheres (positive charge) were modified with the negatively charged samples by electrostatic forces and then attached to an AFM tipless probe (NP-O10, Bruker Inc., Santa Barbara, CA, USA) using a UV-curable adhesive. To obtain a sample-coated substrate, the SiO_2_ wafer was first hydrophilized using a mixture of 98 wt% H_2_SO_4_ and 30 wt% H_2_O_2_ (volume ratio of 3:1) for 12 h, then placed in a COS solution for 3 h to obtain a positively charged surface, and finally immersed in a CNC suspension with a negative charge for 6 h to adsorb a layer of CNCs.

To make the results much more accurate, the spring constant and deflection sensitivity of each probe were calibrated. In this work, a trig threshold of 10 nm in relative trigger mode, a ramp size of 200 nm, and a ramp rate of 1 Hz were set for all force curve measurements. Since the AFM force curve measurements were sensitive and susceptible to external factors, each measurement was carried out 100 times at 10 different locations of the sample-coated substrate to eliminate the error.

### 3.6. Atomistic Molecular Dynamics Simulations

Atomistic molecular dynamics (MD) simulations were performed using the GROMACS (version 2020.6) simulation package [24]. The CHARMM 36 force field and TIP3P water model were used to represent the cellulose molecules and water molecules, respectively [25]. The CNC structure was built through the Cellulose-Builder toolkit according to the Iβ structure [26]. The fibril contains 40 glucoses repeat units and 6 × 6 chains with the long axis along the *z*-axis according to the work of Paavilainen et al. [19]. The fibril was then placed in a 6.5 nm × 6.5 nm × 24.0 nm rectangular box and solvated in water after thousands of steps of energy minimization. Equilibration runs of 5 ns with heavy atom restraint were performed before the production runs of 50 ns without restraints. The resultant structure was replicated in the × direction for the fibril dimer simulation, which extended another 70 ns. The equilibrium dimer structure was used for the investigation of distance and cross-angle relationship, during which one fibril of the dimer was moved for a certain distance or rotated for a small angle to check the energy change. The temperature was coupled to 298 K using the nose-hoover method and the pressure was coupled to 1 atm using the Parrinello–Rahman method. An integration time step of 2 fs was used and the cutoff scheme of 1.2 nm was implemented for the non-bonded interactions. The Particle Mesh Ewald method with a Fourier spacing of 0.1 nm was applied for the long-range electrostatic interactions [27]. All covalent bonds with hydrogen atoms were constrained using the LINCS algorithm [28].

### 3.7. Characterizations

AFM images were acquired on a Multimode AFM Nanoscope-VIII from Bruker (Santa Barbara, CA, USA) using an AFM probe (SNL-10, Bruker Inc., Santa Barbara, CA, USA) with resonance frequency (f_0_) of 56 kHz and spring constant (k) of 0.24 N/m in PeakForce™ ScanAssyst mode at room temperature. The electrophoretic mobility of the CNC suspension and all mixtures was measured using a zeta potential (ζ-potential) analyzer (SZ-100Z, HORIBA, Kyoto, Japan). A quartz crystal microbalance with dissipation (QCM-D, QE401-F1814, Biolin Scientific, Gothenburg, Sweden) was used to quantify the adsorption amount of PAAS and COS on the CNC surface, which was realized by measuring the oscillatory resonant frequencies of a quartz crystal, both fundamental frequency (5 MHz) and selected overtones (15, 25, 35, 45, and 55 MHz). Rheological measurements were performed on a DHR-3 rheometer (TA instrument, New Castle, DE, USA) with a plate geometry (parallel plate diameter of 25 mm, gap width of 1 mm) at 25 °C. The structural coloration of the films was analyzed using UV-visible spectroscopy (UV-2600, SHIMADZU, Kyoto, Japan). POM images were obtained through a polarized light microscope (BX53M, Olympus, Tokyo, Japan). Field-emission scanning electron microscopy (FE-SEM, Merlin, Zeiss, Oberkochen, Germany) was used to observe the microstructure of the film cross-sections.

## 4. Conclusions

In conclusion, this study reported that PAAS and COS with opposite charges have different micro-regulatory mechanisms on CNC self-assembly through a novel collaborative approach of AFM force, MD, and QCM. PAAS tended to converge outside the crystallites instead of being dispersed between CNCs and presented stronger repulsive force than that between CNCs, which compressed the space and reduced the spacing of CNCs. COS was adsorbed to the CNC surface through charge attraction to reduce the surface charge of CNCs, thus reducing the repulsion between CNCs, which also reduces the spacing between CNCs. Then, the decrease in the spacing of CNCs induced the increase of the stacking angle of CNCs and further reduced the number of twisted CNC layers per spiral period. These variations eventually led to the decrease in the pitch of the helix structure of CNC photonic crystals, which allowed the structural color of the final films to exhibit a blue-shift tendency, and even a colorless tendency, after adding 5.0 wt% PAAS as well 3.0 wt% COS. It was confirmed that CNC/PAAS_5.0_ and CNC/COS_3.0_ mixtures with higher concentrations of CNC contributed to maintaining the nematic liquid crystal structure without chirality, wherein the gelation concentrations were 9.0 wt% and 6.0 wt%, respectively. This phenomenon was correlated with kinetic arrest before forming the chiral nematic structure, which prevented gelatinous CNC from further forming a chiral arrangement and finally resulted in the absence of color after water evaporation. In other words, for CNC liquid crystal structure, the synergistic effect of interparticle interaction and twisting of CNCs affected the pitch, while the competitive effect of phase separation and gelation determined the formation of the chiral nematic structure. Overall, this study systematically revealed the influence of charge on the CNC particles’ trajectory at the micro level during the self-assembly process. We believe that this work will bring more inspiration in the controllable and flexible customization of CNC optical structures and CNC-derived photonic materials which could be widely used for structural color encryption and anti-counterfeiting, liquid crystal structural color for producing textiles, sensor devices, and so on. In addition, subsequent work may be able to construct a more accurate model so that the influence of external factors on the self-assembly process can be more precisely determined or even quantitatively analyzed.

## Figures and Tables

**Table 1:** ζ-potential, limit distance, *κ*, and *Z* of samples.

Sample	PAAS-CNC	CNC-CNC	CNC/COS_1.0_- CNC/COS_1.0_	CNC/COS_1.5_- CNC/COS_1.5_	CNC/COS_2.0_- CNC/COS_2.0_	CNC/COS_2.5_- CNC/COS_2.5_	CNC/COS_3.0_- CNC/COS_3.0_
ζ-potential ^a^ (mV)	−56.6 (−38.5)	−37.7 (−37.7)	−34.6 (−34.6)	−31.8 (−31.8)	−29.2 (−29.2)	−28.0 (−28.0)	−26.5 (−26.5)
Limit distance ^b^ (nm)	97.23 (±5.78)	94.43 (±7.56)	91.59 (±3.79)	89.96 (±7.92)	83.31 (±5.17)	78.48 (±5.79)	68.91 (±6.97)
*κ* (nm^−1^)	0.03966	0.04125	0.03823	0.03980	0.04416	0.05182	0.05844
*Z* (×10^−3^ nN)	11.17	9.305	8.594	8.402	7.527	6.504	6.277

^a^ Values outside and inside brackets refer to the ζ-potential of the samples on the probe and on the base, respectively. ^b^ Values in brackets present error limits.

## Data Availability

The data presented in this study are available on request from the corresponding author.

## Supplementary Materials

The following supporting information can be downloaded at: https://www.mdpi.com/article/10.3390/molecules28041857/s1, Figure S1. (a) AFM images of CNCs, and (b) the length distribution and (c) height distribution, respectively; Figure S2. Zeta potential and the Tyndall effect of (a) CNC/PAAS mixtures and (b) CNC/COS mixtures, respectively; Figure S3. The optical photographs (top left), POM patterns (top right) and SEM images (below) of (a) CNC/PAAS films (the ratio of PAAS is 2.0 wt%, 4.0 wt% in order, from left to right), and (b) CNC/COS films (the ratio of COS is 1.5 wt%, 2.5 wt%).; Figure S4. SEM images of (a) the amino silica microspheres, (b) the CNC/PAAS-modified silica microsphere, and (c) the CNC/COS-modified silica microsphere. (d) AFM images of CNC/COS-coated substrate; Figure S5. The force-distance curves and the DLVO fitting curves of (a) PAAS-CNC, CNC/COS-CNC/COS with (b) 1.0 wt%, (c) 1.5 wt%, (d) 2.0 wt%, (e) 2.5 wt%, and (f) 3.0 wt% COS addition, and (g) PAAS-PAAS, respectively; Figure S6. Limit distance of the interaction of different groups of samples based on the fitted curves. (a) CNC-CNC. CNC/COS-CNC/COS with (b) 1.0 wt%, (c) 1.5 wt%, (d) 2.0 wt%, (e) 2.5 wt%, and (f) 3.0 wt% COS addition, respectively. (g) PAAS-CNC; Figure S7. Cross-sectional view of the dimer structural equilibrium process; Figure S8. Side view of the dimer structural equilibrium process; Figure S9. The steady viscosity of CNC/PAAS mixtures at (a) 3.0 wt%, (b) 4.5 wt%, and (c) 7.5 wt% concentration, respectively. The steady viscosity of CNC/COS mixtures at (d) 3.0 wt%, (e) 4.5 wt%, and (f) 9.0 wt% concentration, respectively; Figure S10. (a)–(d) Dependence of G′ and G″ as a function of the angular frequency for CNC/PAAS mixtures with 1.0 wt%–4.0 wt% PAAS addition. (e)–(f) Dependence of G′ and G″ as a function of the angular frequency for CNC/COS mixtures with 1.0 wt% and 1.5 wt% COS addition, respectively.

## Sample Availability

Samples of the compounds are available from the authors.
