# Supplementary material for: Regulatory Mechanism of Opposite Charges on Chiral Self-Assembly of Cellulose Nanocrystals
Source: Molecules. 2023 Feb 15;28(4):1857. doi: 10.3390/molecules28041857 (PMC9959273; doi:10.3390/molecules28041857)
Supplement: Supplementary file 1 [file molecules-28-01857-s001.zip › molecules-2180535-supplementary.pdf]

Supplementary Material

Regulatory mechanism of opposite charges on chiral self-assembly of cellulose nanocrystals

Bin Wang [a], Jinyang Xu [a], Chengliang Duan [a], Jinpeng Li [a, b, *], Jinsong Zeng [a], Jun Xu [a], Wenhua Gao [a], and Kefu Chen [a]

[a] Plant Fiber Material Science Research Center, State Key Laboratory of Pulp and Paper Engineering, School of Light Industry and Engineering, South China University of Technology, Guangzhou 510640, China

[b] Guangdong Provincial Key Laboratory of Plant Resources Biorefinery, Guangzhou, 51006, China

Corresponding Authors

** Name. E-mail: *Jinpeng Li. E-mail: ljp@scut.edu.cn.*

Supplementary Figures

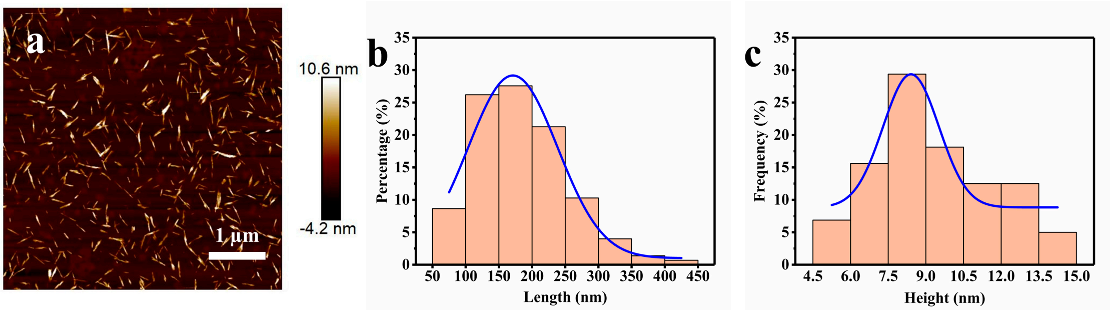

Figure S1. (a) AFM images of CNCs, and (b) the length distribution and (c) height distribution, respectively.

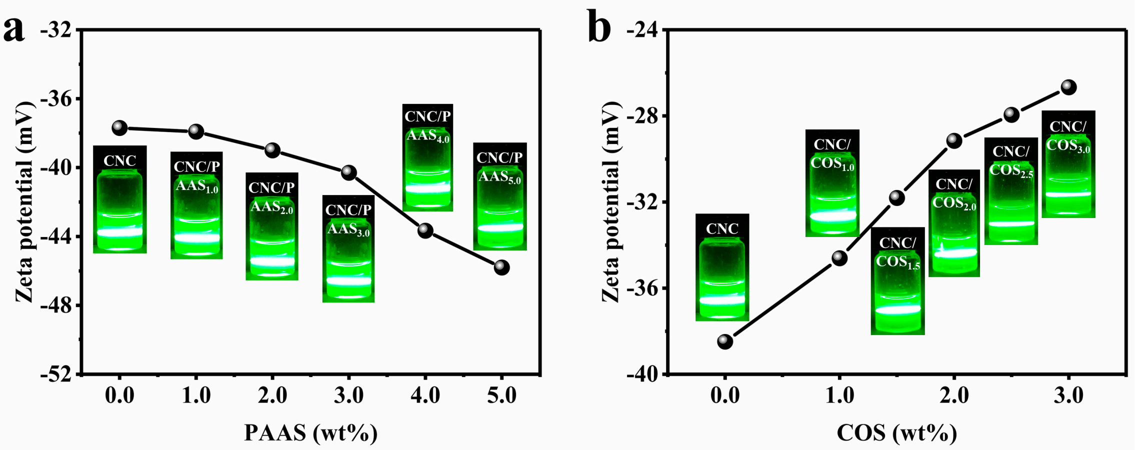

Figure S2. Zeta potential and the Tyndall effect of (a) CNC/PAAS mixtures and (b) CNC/COS mixtures, respectively.

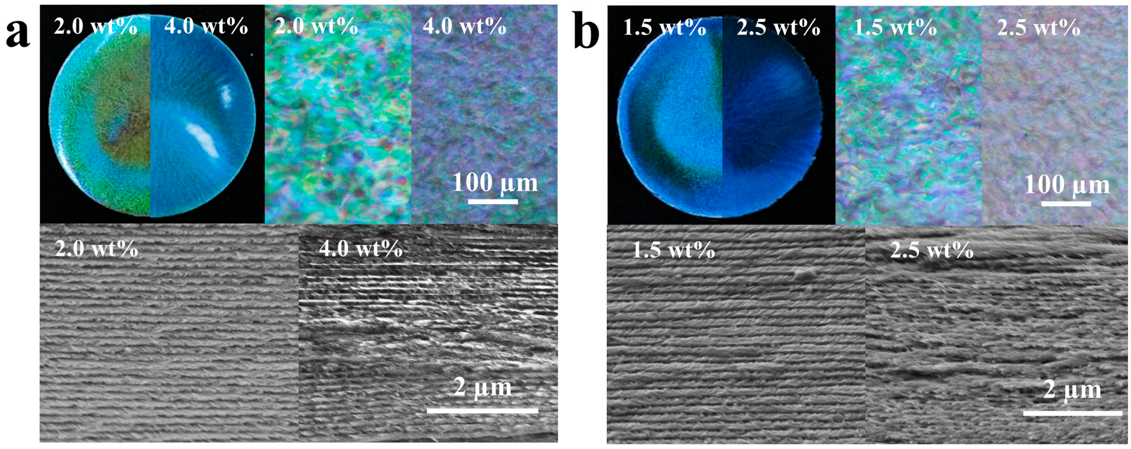

Figure S3. The optical photographs (top left), POM patterns (top right) and SEM images (below) of (a) CNC/PAAS films (the ratio of PAAS is 2.0 wt%, 4.0 wt% in order, from left to right), and (b) CNC/COS films (the ratio of COS is 1.5 wt%, 2.5 wt%).

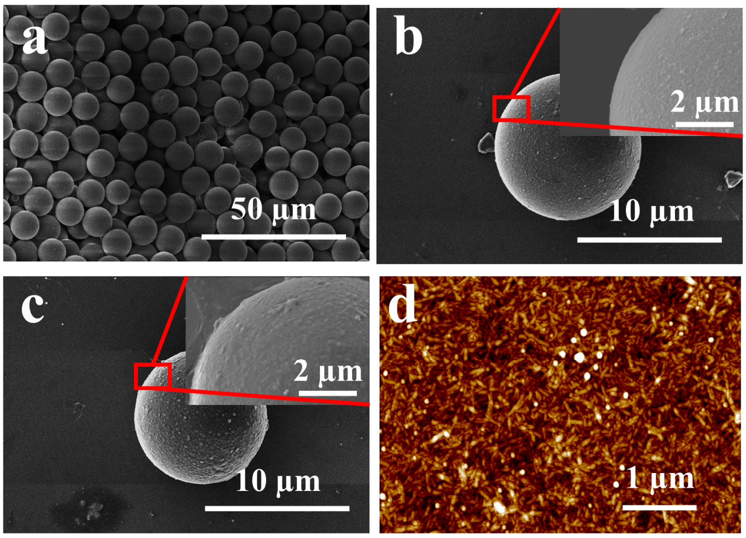

Figure S4. SEM images of (a) the amino silica microspheres, (b) the CNC/PAAS-modified silica microsphere, and (c) the CNC/COS-modified silica microsphere. (d) AFM images of CNC/COS-coated substrate.

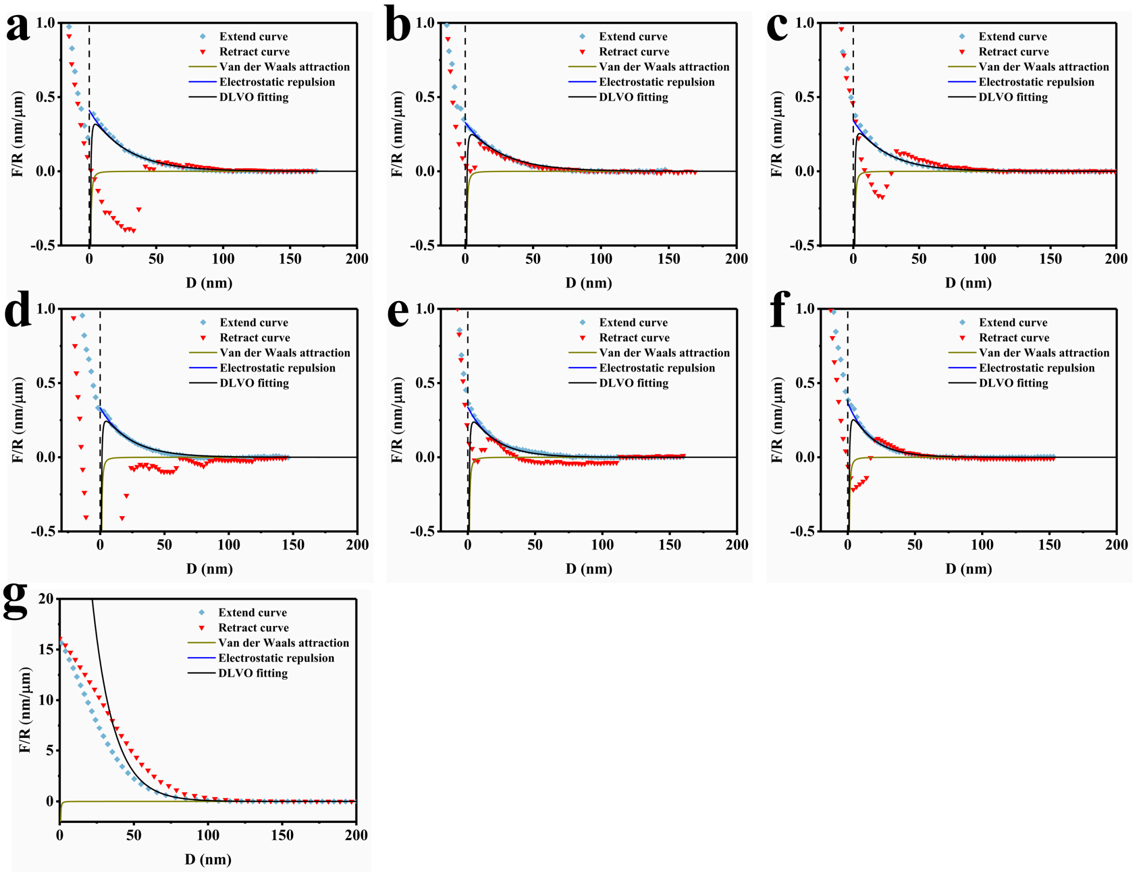

Figure S5. The force-distance curves and the DLVO fitting curves of (a) PAAS-CNC, CNC/COS-CNC/COS with (b) 1.0 wt%, (c) 1.5 wt%, (d) 2.0 wt%, (e) 2.5 wt%, and (f) 3.0 wt% COS addition, and (g) PAAS-PAAS, respectively.

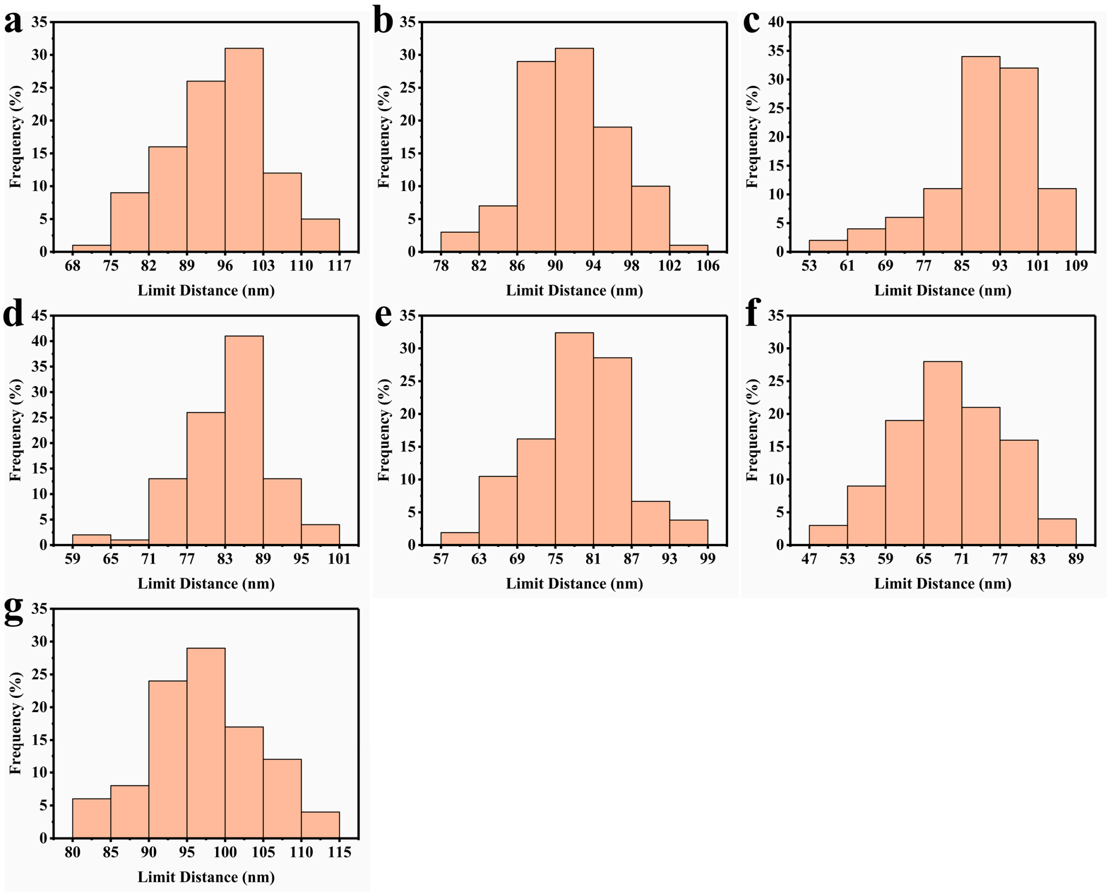

Figure S6. Limit distance of the interaction of different groups of samples based on the fitted curves. (a) CNC-CNC. CNC/COS-CNC/COS with (b) 1.0 wt%, (c) 1.5 wt%, (d) 2.0 wt%, (e) 2.5 wt%, and (f) 3.0 wt% COS addition, respectively. (g) PAAS-CNC.

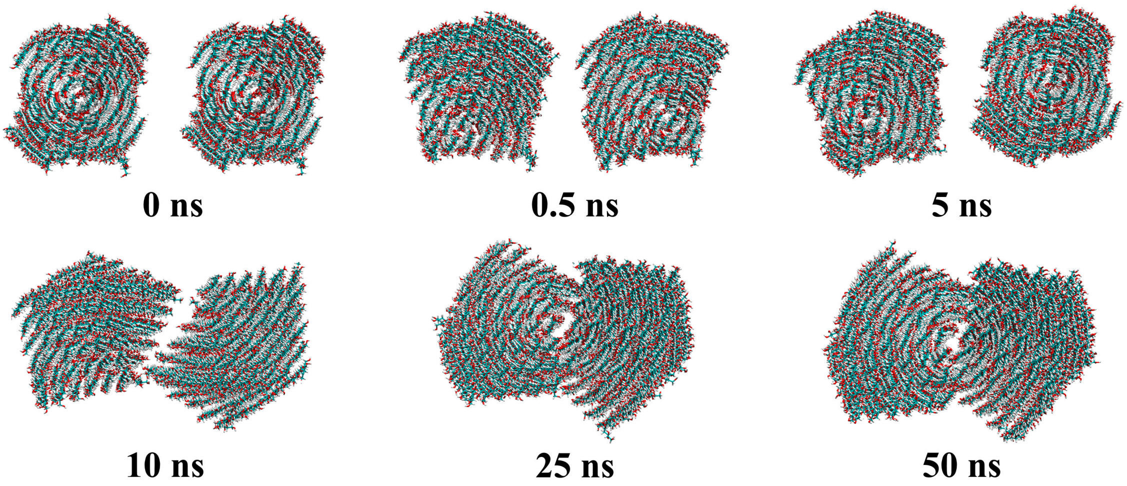

Figure S7. Cross-sectional view of the dimer structural equilibrium process.

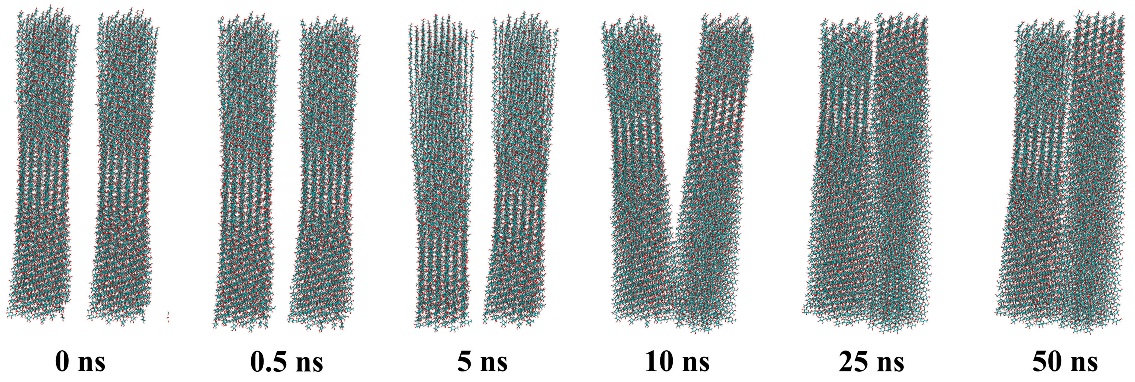

Figure S8. Side view of the dimer structural equilibrium process.

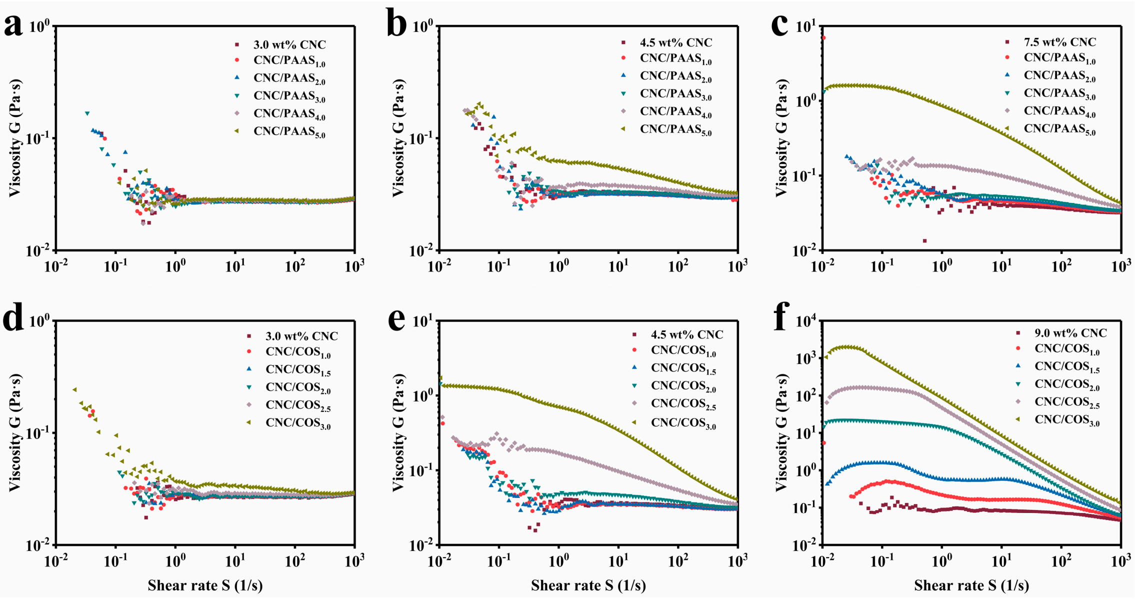

Figure S9. The steady viscosity of CNC/PAAS mixtures at (a) 3.0 wt%, (b) 4.5 wt%, and (c) 7.5 wt% concentration, respectively. The steady viscosity of CNC/COS mixtures at (d) 3.0 wt%, (e) 4.5 wt%, and (f) 9.0 wt% concentration, respectively.

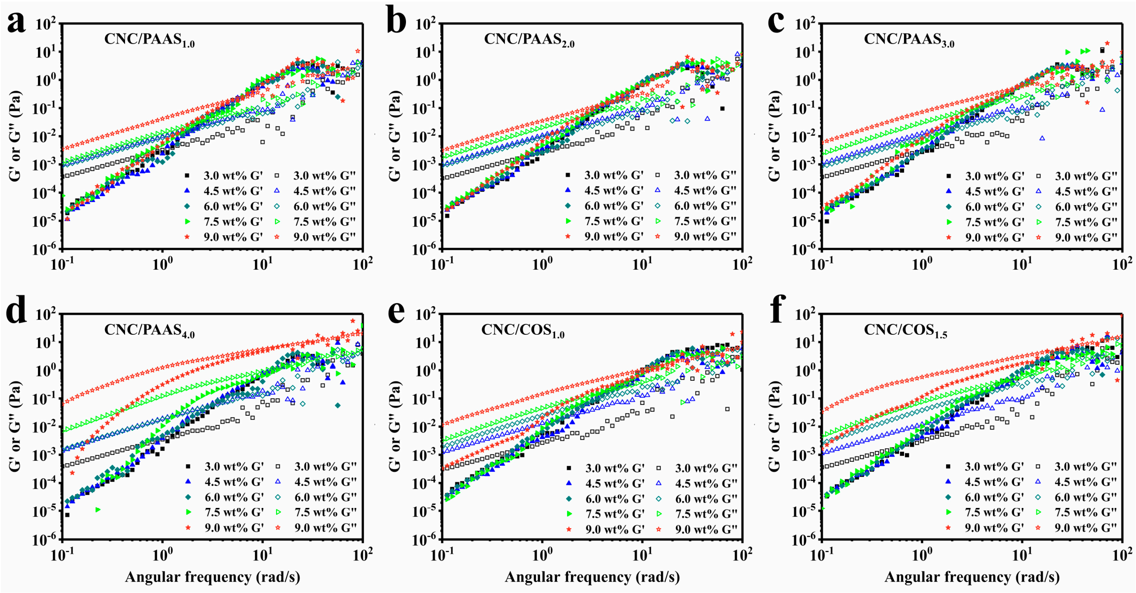

Figure S10. (a)-(d) Dependence of G′ and G″ as a function of the angular frequency for CNC/PAAS mixtures with 1.0 wt%-4.0 wt% PAAS addition. (e)-(f) Dependence of G′ and G″ as a function of the angular frequency for CNC/COS mixtures with 1.0 wt% and 1.5 wt% COS addition, respectively.
